# Screening for Natural Inhibitors of Topoisomerases I from *Rhamnus davurica* by Affinity Ultrafiltration and High-Performance Liquid Chromatography–Mass Spectrometry

**DOI:** 10.3389/fpls.2017.01521

**Published:** 2017-09-01

**Authors:** Guilin Chen, Mingquan Guo

**Affiliations:** ^1^Key Laboratory of Plant Germplasm Enhancement and Specialty Agriculture, Wuhan Botanical Garden, Chinese Academy of Sciences Wuhan, China; ^2^Graduate University of Chinese Academy of Sciences Beijing, China; ^3^Sino-Africa Joint Research Center, Chinese Academy of Sciences Wuhan, China

**Keywords:** topoisomerases I, *Rhamnus davurica*, ultrafiltration, high performance liquid chromatography-mass spectrometry, flavonoids

## Abstract

Topoisomerase I (Topo I) catalyzes topological interconversion of duplex DNA during DNA replication and transcription, and has been deemed as important antineoplastic targets. In this study, the fraction *R.d*-60 from ethyl acetate extracts of *Rhamnus davurica* showed higher inhibitory rates against SGC-7901 and HT-29 compared with the *R.d*-30 fraction *in vitro*. However, the specific active components of *R.d*-60 fraction remain elusive. To this end, a method based on bio-affinity ultrafiltration and high performance liquid chromatography/electrospray mass spectrometry (HPLC- ESI-MS/MS) was developed to rapidly screen and identify the Topo I inhibitors in this fraction. The enrichment factors (EFs) were calculated to evaluate the binding affinities between the bioactive constituents and Topo I. As a result, eight ligands were identified and six of which with higher EFs showed more potential antitumor activity. Furthermore, antiproliferative assays *in vitro* (IC_50_ values) with two representative candidates (apigenin, quercetin) against SGC-7901, HT-29 and Hep G2 cells were conducted and further validated. Finally, the structure-activity relationships revealed that flavones contain a C2-C3 double bond of C ring exhibited higher bio-affinities to Topo I than those without it. This integrated method combining Topo I ultrafiltration with HPLC-MS/MS proved to be very efficient in rapid screening and identification of potential Topo I inhibitors from the complex extracts of medicinal plants, and could be further explored as a valuable high-throughput screening platform in the early drug discovery stage.

## Introduction

In the early drug discovery stage of small molecules, the binding affinity between small molecular candidate and its therapeutic biomolecule targets is considered as the primary determinant of the candidate’s biological activity (Qin et al., 2015). Recently, it has been found that almost half of the small-molecule drugs in the market are enzyme inhibitors according to a survey (Shi et al., 2013). As we know, a lot of medicinal plants (such as Traditional Chinese Herbal Medicines, TCM) have been used as medicines or food supplements for the treatment of a wide variety of diseases in China and some other Asia countries for quite a long time (Xiao S. et al., 2015). In consideration of very diversified chemical structures and biological activities of plant secondary metabolites, medicinal plants have become a promising medicinal resources with their natural active components. Nevertheless, a single herb may contain numerous compounds and it is complicated to figure out the correlations between those complex constituents and their corresponding biological activities (Zhu et al., 2013). Consequently, it is extremely important to develop an efficient strategy for rapid screening and identification of those bioactive compounds responsible for their pharmacological effects from medicinal plants. Furthermore, it will also offer new opportunities for the discovery of some novel therapeutic agents from natural resources.

*Rhamnus davurica* Pall. (Rhamnaceae) has long been traditionally consumed as a kind of folk remedy in China and other Asian countries. The medicinal parts of *R. davurica* are the barks, leaves and seeds, which have been proved to possess many pharmaceutical activities for the treatment of dysuresia, pruritus, constipation and allergic diseases, etc. (Kim et al., 2015). Mai et al. (2001) revealed that the ethyl acetate (EA) extracts from the fruits of *R. nepalensis* Laws., obtained in Vietnam, showed significant cytotoxicity to the KB cell line. Wei et al. (2000) also reported that the isolated flavonoids like quercetin, kaempferol, and quercetin 3-*O*-methyl ether from *R. nakaharai*, and frangulin B from *R. formosana*, exhibited remarkable anti-inflammatory effects on the chemical mediators released from neutrophils, mast cells, microglial cells and macrophages. Several types of constituents, including flavonoids (aglycones of kaempferol, myricetin, quercetin, isorhamnetin), anthraquinones (emodin, aloe-emodin, physcion, chrysophanol), naphthols, anthrone, triterpenes and their glycosides have been isolated and identified from *Rhamnus* species (Berhanu and Martin, 1995; Mohamed et al., 1999; Mai et al., 2001; Sharp et al., 2001; Chen et al., 2016a). Nevertheless, the antineoplastic effects of *R. davurica* still remain unclear up to now. Moreover, most of the current pharmacological studies on this herb medicine mainly focused on its crude extracts, the actual bioactive constituents responsible for the pharmacological activity in the extracts are still unknown. In addition, little work has been conducted to rapidly screen and identify the bioactive constituents from the crude extracts, and evaluate the degrees of interaction between these active components and their corresponding pharmacological effects. Hence, rapid screening and identification of these active components could be essential to further understand its pharmacological effects.

DNA topoisomerases are nuclear enzymes and widely found in prokaryotic and eukaryotic cells. They catalyze the interconversion of topological isomers of DNA molecules, and play a crucial part in the consecutive breakage and reunion of DNA strands during DNA synthesis (Chowdhury et al., 2002). On the other hand, topoisomerase inhibitors have long been considered as potential anti-cancer drug candidates. Depending on the different mechanisms of action, there exist two classes of DNA topoisomerases: topoisomerase I (Topo I) and topoisomerase II (Topo II). Unlike the Topo II acting at both strands of DNA, Topo I does not require ATP hydrolysis and acts as the DNA-metabolizing enzyme required for the rNMPs (ribonucleoside monophosphates)-associated deletion signature (Kim et al., 2011; Chimento et al., 2015), which catalyzes topological interconversion in duplex DNA by reversibly breaking and rejoining one strand during many pivotal cellular processes such as transcription, replication, and chromosome condensation (Nino et al., 2007). Therefore, inhibitors of Topo I, which can block the DNA synthesis during malignant cell proliferation, are considered as important targets of antineoplastic agents with the mechanism of DNA interaction (Li and Liu, 2001). It is reported that camptothecin (CPT), a very famous natural drug, has its unique cellular target receptor as Topo I and displays significant anticancer effect (Majumdar et al., 2015; Schovanek et al., 2015). What’s more, the two CPT derivatives-topotecan (TPT) and irinotecan (CPT-11), have already become the only Topo I inhibitors approved by the FDA for the treatments of ovarian, colorectal and lung cancers (Chaudhuri et al., 2012).

The conventional approaches for screening the natural-origin bioactive compounds require multiple-step isolations, which are labor-intensive, and time-consuming with relatively high risk of failure (Nakai et al., 2005; Zhang et al., 2013; Xiao S. et al., 2015). In order to obtain both structural and bioactivity information in a high-throughput screening in recent years, a good combination of the affinity ultrafiltration and high performance liquid chromatography coupled with electrospray mass spectrometry (HPLC-ESI-MS/MS) has been developed to identify numerous interesting and/or novel compounds without tedious prior isolation, provides pivotal insights into biomolecule structures and ligands binding properties (Qin et al., 2015), and meanwhile illustrates the potential biological mechanisms (Katoch et al., 2012). In a similar way, we can simplify the screening and identification of the targeted constituents from natural products by combining affinity ultrafiltration with HPLC-MS/MS (UF-HPLC-MS) (Tao et al., 2015). In this assay, the bio-affinity ultrafiltration separates the ligand-receptor complexes from unbound compounds, and the ligands released from the complexes could be readily identified and subsequently quantified by LC-MS/MS analysis. Thus, UF-HPLC-MS possessed several obvious advantages, including but not limited to the recycling of enzymes, no need for fixation, and low sample consumption, in the receptor-ligand interaction study, and played an increasingly important role for the high-throughput screening and identification of bioactive compounds from complex mixtures in the early drug discovery stage (Li et al., 2015; Qin et al., 2015; Xiao S. et al., 2015). Additionally, the antagonistic or synergistic effects among those compounds may also be investigated.

Our previous *in vitro* screening study has demonstrated that the EA extracts of *R. davurica* showed significant inhibitory effects on human cell lines of SGC-7901 (gastric carcinoma) and HT-29 (colon carcinoma) with the inhibition rate of 43.64 and 45.53%, respectively. Nevertheless, it is still unclear that which constituents are the major active ingredients against cancer. To this end, a simple and rapid screening assay based on the UF-HPLC-MS method was developed to screen and identify Topo I inhibitors in the complex extracts from *R. davurica* in this study. Prior to the UF-HPLC-MS, the polyamide column was used to separate the EA extracts of *R. davurica* into two fractions, followed by the antiproliferative activities against SGC-7901 and HT-29 *in vitro*. Results showed that eight Topo I inhibitors were discovered and identified from the extracts of *R. davurica*, and six of them exhibited higher enrichment factors (EFs), which means more potential antitumor activity. Additionally, further *in vitro* experiments of candidate inhibitors against SGC-7901, HT-29, and Hep G2 (liver cancer) cells were conducted to validate the screening method, and further the anti-proliferative effects. The combination of the UF-HPLC-MS with representative drug targets (such as Topo I) offers a powerful tool to rapidly screen and identify bioactive compounds from the complex mixture such as crude extracts from medicinal plants.

## Materials and Methods

### Materials, Chemicals and Reagents

The barks of *R. davurica* Pall. (growing in China’s northeast) were purchased from Jikang pharmaceutical Co., Ltd. (Hebei, China). The oven-dried samples were ground using a domestic blender, and packed in sealed polyethylene bags, then stored in a refrigerator at 4°C until use. The authentication and identification of the specimen was kindly assisted by our taxonomist (Professor Guangwan Hu) at the Key Laboratory of Plant Germplasm Enhancement and Specialty Agriculture, Wuhan Botanical Garden, Chinese Academy of Sciences. A voucher specimen (No. 0031) was deposited in herbarium of the Key Laboratory.

The reference standards of apigenin, quercetin and camptothecin were purchased from Shanghai Tauto Biotech (Shanghai, China) and Aladdin Industrial Corporation (Shanghai, China). Formic acid, methanol and acetonitrile (ACN) of HPLC grade were purchased from TEDIA Company Inc. (Fairfield, OH, United States). Polyamide was obtained from an industrial chemical company affiliated to Nan Kai University (Tianjin, China). DNA Topo I was purchased from NEW ENGLAND Biolabs (Beijing) Inc. (Beijing, China), and centrifugal ultrafiltration filters (YM-30, 30 kDa) were provided by Millipore Co. Ltd. (Bedford, MA, United States). Water for HPLC and LC-MS/MS was prepared with EPED (Nanjing Yeap Esselte Technology Development Co., Nanjing, China). All other chemicals and solvents were of analytical grade.

### Preparation of the Two Fractions of *Rhamnus davurica*

Firstly, 100 g raw powder sample of *R. davurica* was accurately weighted and then extracted in an ultrasonic bath with 60% ethanol for 30 min at room temperature, and the residue was re-extracted twice as described above. After being filtered by a quantitative filter paper under reduced pressure, the combined filtrates were concentrated in a rotary evaporator under reduced pressure at 40°C to afford the crude syrup extract. Later, the crude extract was dispersed in water (100 mL) and subjected to liquid-liquid fractionation with petroleum ether (PE, b.p. 60–90°C, to remove chlorophyll), and ethyl acetate (EA), successively. Afterward, a part of EA fraction was eluted on a polyamide column (45 cm × 5.3 cm) with distilled water to nearly colorless, and subsequently with 30 and 60% ethanol to give the two main fractions (*R.d*-30 and *R.d*-60), respectively. Finally, the two fractions were lyophilized in a freeze dryer to dryness for the subsequent analysis.

### Antiproliferation Assays

The antiproliferation assays using two human cell lines: SGC-7901 (gastric carcinoma) and HT-29 (colon carcinoma) were performed according to our previous study with slight modification (Tian et al., 2015). Briefly, both cell lines were seeded into a 96-well plate at a density of 5000 cells in DMEM (Dulbecco’s Modified Eagle Medium), supplemented with 10% fetal bovine serum (FBS), 1% penicillin- streptomycin and incubated in a humidified atmosphere containing 5% CO_2_ at 37°C for 24 h, respectively. The fractions of *R.d*-30 and *R.d*-60 were dissolved in dimethyl sulfoxide (DMSO), then diluted with the medium, and further dispensed into each well to a final concentration of 150 μg/mL. The solvent, DMSO, was used as the blank control. After incubating mixtures of cells and tested fractions for 72 h, 20 μL of MTT [3-(4,5-dimethyl-2-thiazolyl)- 2,5-diphenyl-2-H-tetrazolium bromide] solution (5.0 mg/mL) was added into each well, and incubated for a further 2 h. The optical density (OD) values of the plate were detected using a Tecan plate reader at the excitation wavelength of 492 nm, and emission wavelength of 595 nm. Mean absorbance values of each fraction were calculated and the control values were subtracted from the mean values of the corresponding samples. The inhibition rate (%) was calculated using the equation: Inhibition rate (%) = (ODC – ODT)/ODC × 100%, where ODC and ODT were the OD values of control and fractions of *R. davurica*, respectively. Besides, the activity of camptothecin was also tested as a positive control. Each sample was tested in triplicate, and the results were expressed as means ± SD (standard deviation).

### Screening Potential Topo I Inhibitors with Ultrafiltration

The present procedure was modified according to our most recent work (Chen et al., 2016b). In Brief, 100 μL of tested sample solution (*R.d*-60, 1.0 mg/mL) was mixed with 10 μL Topo I (0.5 U/μL) and incubated for 30 min at 37°C in a 0.2 mL Eppendorf (EP) tube. After that, the mixture was filtered through a 30 kDa molecular weight cut-off ultrafiltration membrane (YM-30), and then centrifuged at 10,000 rpm for 10 min at room temperature. The filtrate was washed three times by centrifugation with 200 μL of NEBuffer (pH 7.9) to remove the unbound compounds. Then, the bound compounds were dissociated by adding 200 μL of ACN/water (90:10, v/v) and then centrifuged at 10,000 rpm for 10 min with three times. The filtrates were combined and dried by lyophilization using a centrifugal evaporator, and later the residue was dissolved in 50 μL 90% aqueous ACN (containing 2.0 μg/mL isoschaftoside as the internal standard) and directly analyzed using HPLC-ESI-MS/MS. Following the similar procedure, inactivated Topo I solution (boiled for 10 min in water bath) was used as the negative control. The relative contents of compounds of interest were calculated based on the peak areas from the HPLC chromatography against that of the added standard (isoschaftoside).

### HPLC-ESI-MS/MS Analysis

The phytochemicals in the *R.d*-60 fraction before and after ultrafiltration were directly analyzed by HPLC-ESI-MS/MS using a TSQ Quantum Access MAX mass spectrometer (Thermo Fisher Scientific, San Jose, CA, United States) equipped with a Thermo Accela 600 HPLC system. The HPLC-UV system consisted of a quaternary pump (1250), on-line vacuum degasser, autosampler and thermostatic column compartment, connected in line to a UV detector prior to the mass spectrometer. A reverse C18 column (4.6 mm × 250 mm, 5.0 μm; Waters Symmetry, United States) was used at a flow rate of 0.6 mL/min. The powder of *R.d*-60 was dissolved in methanol (1.0 mg/mL), and used directly for HPLC-ESI-MS/MS analysis. A 10 μL aliquot of sample solution was injected into the HPLC system, and a binary gradient HPLC conditions were: solvent A (0.1% formic acid-H_2_O), and solvent B (acetonitrile, ACN). The elution procedures were conducted at 30°C and used as follows: 20% B in 0–5 min, 20 to 70% B in 5–30 min, and 70% B in 30–35 min. The UV detection wavelength was set at 360 nm. The ESI-MS/MS experiments were carried out using a TSQ Quantum Access MAX mass spectrometer with an ESI source operating in Auto-MS^n^ mode. The MS^n^ conditions were set as follows: the electrospray capillary voltage, 3,000 V; nebulizing gas flow rate, 6.0 L/min; sheath gas (N_2_) pressure, 40 arbitrary units; Aux gas (N_2_) pressure, 10 arbitrary units; vaporizer temperature, 300°C; capillary temperature, 250°C; collision energy (CE), 10 V; collision energy grad (CE grad), 0.035 V/m. Mass spectrometry data were acquired in the Data-Dependent Scan mode (mass range *m/z* 150–1500). All of MS^n^ analyses were operated in the negative ion (NI) mode, and data acquisition and analysis was performed in the Thermo Xcalibur ChemStation.

## Results

### Antiproliferative Assay on the Two Fractions of *Rhamnus davurica*

**FIGURE 1:**
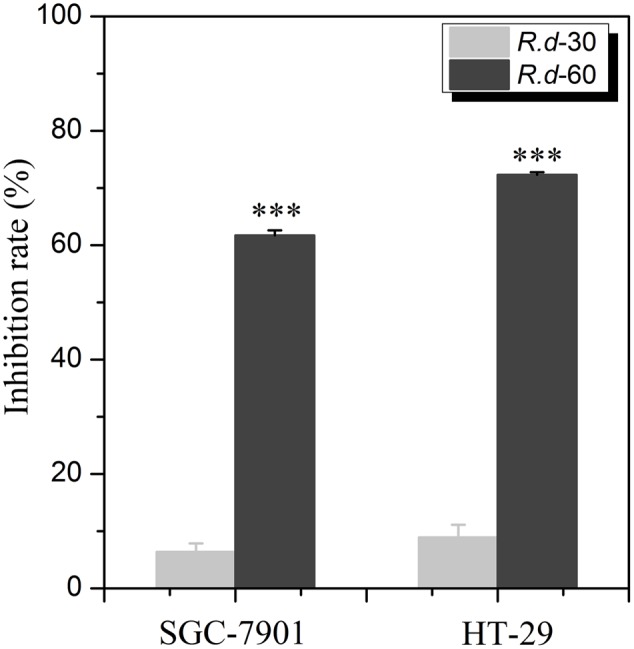
The anti-proliferative activities on two human cancer cell lines with the *R.d*-30 and *R.d*-60 fractions from *Rhamnus davurica*. Statistical analysis of untransformed data was performed using SPSS version 16.0 statistical Software, and results were considered significant at *p* ≤ 0.05 (^∗∗∗^*p* < 0.001); SGC-7901: human cell lines of gastric carcinoma; HT-29: human cell lines of colon carcinoma.

In our previous study, the EA extracts of *R. davurica* were found to exhibit potential anti-proliferative effects on SGC-7901 and HT-29 cells with the inhibition rates at 43.64 and 45.53%, respectively. In this study, we focused on the anti-proliferative fraction of *R. davurica* in order to explore its active components. To this end, the comparisons of the anti-proliferative activity of *R.d*-30/60 on SGC-7901 and HT-29 *in vitro* were conducted. As shown in **Figure 1**, it is found that the *R.d*-60 showed significantly higher inhibitory rates against SGC-7901 and HT-29 at 61.72 and 72.29% (*p* < 0.001) at the same dose, compared with those of the *R.d*-30 at 6.37 and 8.92%, respectively, which indicated the *R.d*-60 was the active fraction and conducive to the further identification of its active compounds.

### Screening Potential Topo I Inhibitors from *Rhamnus davurica* Combining Ultrafiltration with HPLC-MS/MS

**FIGURE 2:**
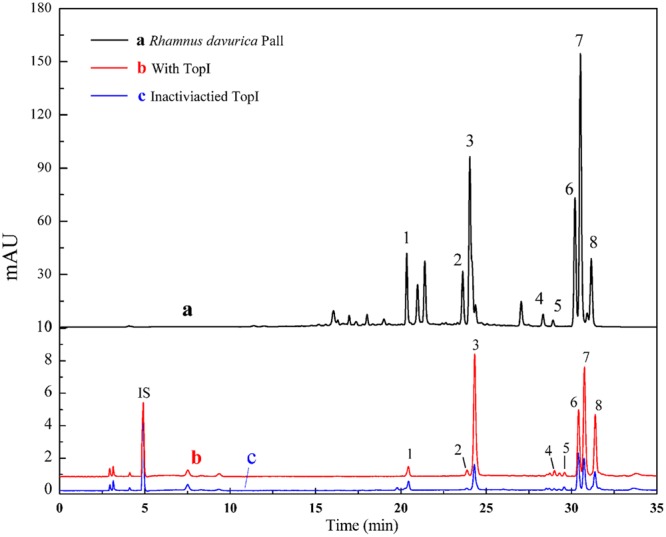
The HPLC chromatograms of the chemical constituents in the *R.d-60* fraction from *R. davurica* obtained by ultrafiltration at 360 nm. The black solid line (a) represents HPLC profiles of the extract of *R.d*-60 fraction without ultrafiltration; and the red line (b) and blue line (c) represent HPLC profiles of the *R.d-60* fraction with activated and inactivated Topo I, respectively. Isoschaftoside was used as the internal standard (IS).

**Figure 2** shows the ultrafiltration-HPLC analysis of the constituents from *R.d*-60 fraction, and some components in this fraction exhibited specific binding to Topo I. Obviously, the chromatogram of *R.d*-60 is very different before and after incubation with Topo I. In total, eight peaks of activated group gave bigger peak areas than the inactivated control group after incubation, which implied these eight compounds showed specific binding toward Topo I and were considered as potential ligands of topoisomerase with different anti-proliferative activities. Meanwhile, the relative contents of the eight peaks from activated and inactivated group were calculated based on the peak areas of the corresponding compounds against that of IS as shown in **Table 1**. It’s interesting to find that the eight compounds from activated group exhibited relatively higher contents than those of the control group incubated with inactivated enzyme. At the same time, other compounds in the *R.d*-60 fraction were not considered as Topo I ligands, which were supposed not to be distinguished by the ultrafiltration screening assay.

**Table 1:** The relative amounts, the enrichment factors (EFs) and the UF-HPLC-MS data of potential inhibitors of Topo I from the *Rd-60* fraction.

Number	*t*_R_ (min)	C (μg/ml)	EF (%)	[M-H]^-^	MS/MS data	Identification
1	20.4	0.33	0.4	287	287, 259, 243, 201, 151, 125	Aromadendrin^b^
2	23.8	0.18	0.7	271	271, 227, 165, 151, 107	Naringenin^b^
3	24.3	4.31	5.2	269	269, 224, 201, 181, 159, 133, 107	Apigenin^a^
4	29	0.16	3.0	301	301, 283, 165, 135, 109	Quercetin^a^
5	29.6	0.11	5.6	299	299, 284, 211, 150	Rhamnocitrin^b^
6	30.4	2.10	1.7	285	285, 270, 243, 165, 119, 93	Sakuranetin^b^
7	30.8	3.63	3.3	283	283, 268, 240, 211, 117	Questin^b^
8	31.4	2.03	5.1	283	283, 268, 239, 211, 195	Physcion^b^
9(IS)	4.9	2.00		563	563, 503, 473, 383, 353	Isoschaftoside

*^a^Compared with the corresponding standard; ^b^Identified based on the published literature; 2 μg·mL^-*1*^ isoschaftoside as the internal standard.*

Based on the variations of the peak areas before and after incubation with Topo I, the EFs can be used to determine the degree of affinity between the ligand and the enzyme. The EF was calculated as follows:

$$\begin{array}{c} \text{EF}(\%)\text{=}\;\frac{\left(\text{At-Ac}\right)}{\text{A0}}\times 100\% \end{array}$$

where At, Ac, and A0 represent the peak areas obtained from incubation of the *R.d-60* fraction with activated, inactivated and without Topo I in **Figure 2** (Zhu et al., 2013), respectively. Among those chemical constituents from *R.d*-60, the unique EF could assess binding affinity between each compound to Topo I, and also imply the characteristic anti-proliferative activity. The results showed that compound 5 possessed the greatest degree of affinity (5.6%), followed by compounds 3 (5.2%), 8(5.1%), 7 (3.3%), 4 (3.0%), and 6 (1.7%) in **Table 1**. As expected, the EFs for each compound were different from each other, and even the lower abundant peak corresponding to the compound 5, for example, exerted a relative higher bio-affinity to Topo I. Therefore, the discrepant EFs may be due to their competitively distinguished interactions with Topo I.

### Identification of Topo I Inhibitors in *R.d*-60 Using HPLC-ESI-MS/MS

**FIGURE 3:**
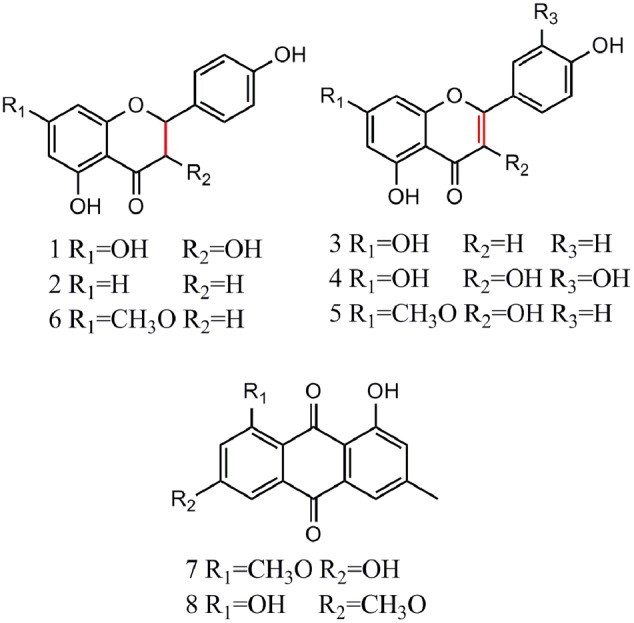
Chemical structures of eight compounds identified from *R.d-60* based on the MS/MS spectra of the corresponding standards.

**FIGURE 4:**
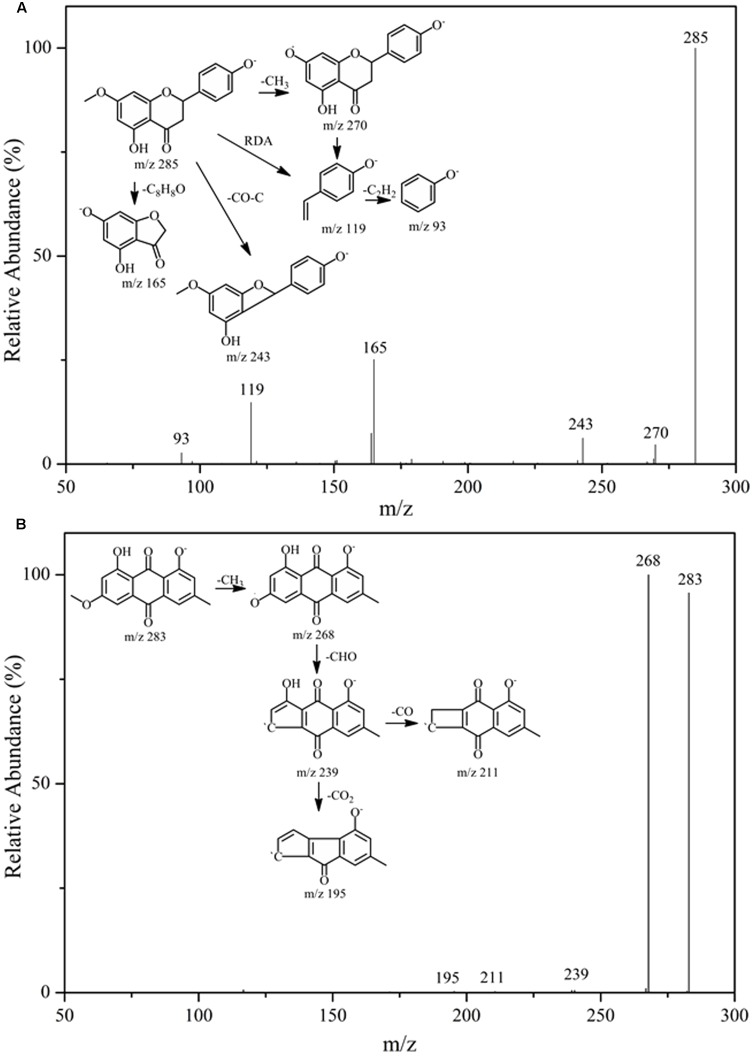
The fragmentation pathways proposed according to the MS/MS spectra of compounds 6 **(A)** and 8 **(B)**.

After incubation with Topo I and ultrafiltration affinity screening, the eight compounds from *R.d*-60 with different affinities to Topo I were identified or characterized by both their HPLC retention time and MS/MS spectra in the negative ion mode. Also, the retention times (*t*_R_), molecular masses, and contents and fragment ions of these eight components are listed in **Table 1**, and their corresponding structures are shown in **Figure 3**. Furthermore, the representative fragmentation pathways of compounds 6 and 8 were also proposed according to their corresponding MS/MS spectra and shown in **Figure 4**.

By comparing the MS/MS data with those of the corresponding standards (Akiyo et al., 1983; Malla et al., 2012; Chirumbolo, 2014), compounds 1, 2, and 6 (ESI-MS: *m/z* 287 [M-H]^-^, 271 [M-H]^-^, 285 [M-H]^-^, respectively) were easily characterized as aromadendrin, naringenin, and sakuranetin, respectively; they commonly shared a same basic parent skeleton of dihydroflavone as shown in **Figure 3**. Interestingly, compounds 3, 4, and 5 (ESI-MS: [M-H]^-^ at *m/z* 269, *m/z* 301, *m/z* 299, respectively) had the same basic parent skeleton of flavone (**Figure 3**) and were identified as apigenin, quercetin and rhamnocitrin according to the corresponding standards and previous study (Mohamed et al., 1999; Sandra and Paula, 2010). Most of the fragment ions for flavone skeleton were also observed by ESI-MS/MS, and further fragment ions could be more informative for structural elucidation. For example, the deprotonated precursor ion of compound 5 at *m/z* 299 generated some fragment ions, such as [M-H-CH_3_]^-^ at *m/z* 284, [M-H-CH_3_-CO_2_-COH]^-^ at *m/z* 211, and *m/z* 150 produced by the RDA-cleavage of C ring. With the same [M-H]^-^ ion at *m/z* 283 in the ESI-MS in the negative ion mode (**Table 1**), compounds 7 and 8 were identified as questin and physcion in accordance with a C_16_H_12_O_5_ formula, considering the retention times, elution profile in C18 columns and the similar collision-induced dissociation (CID) pathways reported for the two isomers (Gauthier et al., 2012; Yan et al., 2014). Additionally, the same ion at *m/z* 268 corresponds to the loss of a methyl moiety (15 Da).

Flavonoids and anthraquinones are widely consumed as food supplements or functional foods from natural products, and have shown remarkable biological or pharmacological activities (Chowdhury et al., 2002). In **Figure 3**, six flavonoids could be classified into two subtypes, dihydroflavones and flavones, and two anthraquinones were detected in the *R.d-60* according to their MS/MS spectra after UF-HPLC-MS assay. In order to further elucidate the structures of flavonoids and anthraquinones, the MS/MS spectra of representative compounds 6 and 8 were applied to elaborate the proposed fragmentation pathways as shown in **Figure 4**. Compound 6 was identified as sakuranetin (4′, 5-dihydroxy-7- methoxyflavanone, calculated as C_16_H_14_O_5_, with Mw. 286, **Figure 4A**), which was indicated by a deprotonated molecular ion [M-H]^-^ at *m/z* 285 in the negative ion mode, [M-H-CH_3_]^-^ at *m/z* 270, [M-H-CO-C]^-^ at *m/z* 243, and [M-H-C_8_H_8_O]^-^ at *m/z* 165. Additionally, the ion at *m/z* 119 corresponds to the characteristic RDA-cleavage of C ring, and further to the dissociation of acetenyl moiety (24 Da). By comparison of the proposed fragmentation pathways and the MS/MS spectra with those of previous study, compound 6 was suggested as sakuranetin (Chirumbolo, 2014). For compound 8 ([M-H]^-^ at *m/z* 283), several fragment ions at *m/z* 268, 239, 211, and 195 were obtained. Fragmented ions of [M-H-15]^-^ at *m/z* 268, [M-H-15-29]^-^ at *m/z* 239 and [M-H-15-29-44]^-^ at *m/z* 195 were produced by the successive loss of methyl moiety (15 Da), neutral CO+H moiety (29 Da) and CO_2_ moiety (44 Da). Besides, the fragment ion at *m/z* 211 implied a neutral loss of CO moiety (28 Da) from [M-H-CH_3_-COH]^-^ at *m/z* 239. As a result, compound 8 was deduced to be physcion (1,8-dihydroxy-3-methoxy-6-methyl-9,10- anthracenedione, calculated as C_16_H_12_O_5_, Mw. 284, **Figure 4B**), whose fragmented ions were also consistent with those observed using ESI-MS/MS (Yan et al., 2014).

### Determination of the Half-Maximal Inhibitory Concentrations (IC_50_ Values)

In this study, two representative bioactive compounds (3 and 4) with higher EFs were investigated against SGC-7901, HT-29, and Hep G2 cells in a concentration range of 0.37–30.0 μg/mL to further validate the screening method and the potential anti-proliferative effects. Camptothecin was served as the positive control, which was the first small molecule identified as a Topo I inhibitor (Majumdar et al., 2015). The assay results were listed in **Table 2**. It was observed that compounds 3 and 4 showed notably inhibition to proliferation of all of the three cancer cells tested.

**Table 2:** The half-maximal inhibitory concentrations (IC_50_values) of pure compounds on human cancer cell lines.

Number	Compound	IC_50_ (μg/mL)		
		SGC-7901	HT-29	Hep G2
3	Apigenin	17.76 ± 0.76	19.79 ± 0.32	10.20 ± 0.24
4	Quercetin	12.70 ± 0.71	29.74 ± 0.38	4.78 ± 0.28

*The IC_50_ values of the pure compounds screened from *Rhamnus davurica* on human cell lines of SGC-7901 (gastric carcinoma), HT-29 (colon carcinoma), and Hep G2 (liver cancer).*

**FIGURE 5:**
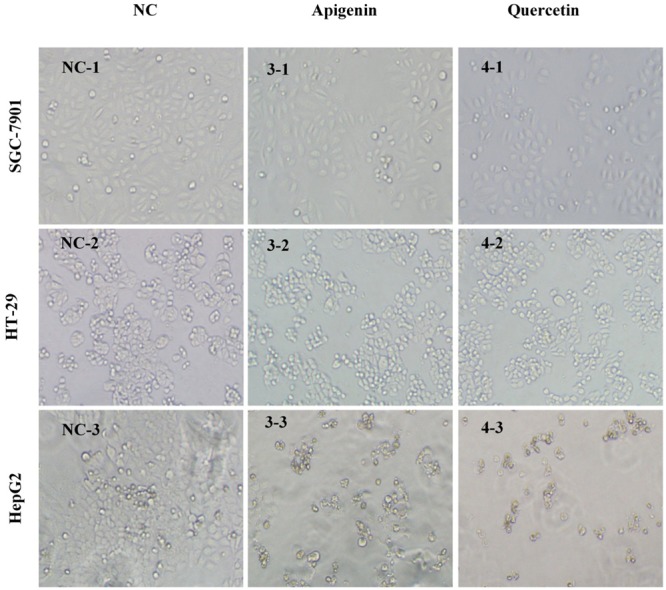
Morphological illustrations of SGC-7901, HT-29 and Hep G2 cell populations treated with apigenin and quercetin for 72 h (100×). The illustrations demonstrate the strong growth inhibitory activity of the two compounds using phase-contrast microscopy. Images are from a representative test, and the concentrations here approximately equal to the IC_50_ values of corresponding cancer cell lines acquired in the anti-proliferative assay *in vitro*, respectively. NC, Normal Control; 3-, peak 3 (apigenin); 4-, peak 4 (quercetin); -1, SGC-7901; -2, HT-29; -3, Hep G2.

At the same time, cell populations and morphological changes were observed with a phase-contrast microscopy after treated with apigenin and quercetin for 72 h. The concentrations here were approximately equal to the IC_50_ values of corresponding cancer cell lines acquired in the anti-proliferative assay *in vitro*, respectively. Two compounds mentioned above effectively inhibited cell proliferation in a concentration-dependent manner, which were also in accordance with the previous MTT results. Meanwhile, significant reduction of viable cells caused by the drug treatment was observed using microscopic observations as shown in **Figure 5**. Furthermore, cells treated with apigenin and quercetin showed distinct morphological changes, including cell shrinkage, decreased intercellular adhesion, scattering and expanded intercellular spaces compared with those untreated cells. In the early stages of apoptosis, many morphological changes of apoptotic features, such as cell shrinkage, membrane blebbing and so on, occurred commonly in HCT-116, Hep G2, and ovarian cells (Lee et al., 2014; Yi et al., 2014; Zhao et al., 2014). Intervention with apigenin and quercetin, caused significant pharmacodynamic effects on the cellular morphology of those cancer cells, which were very similar to the results of the previous studies (Wei et al., 1990; Casagrande and Darbon, 2001; Zhao et al., 2014).

## Discussion

### Screening for Topo I Inhibitors in *R.d*-60

The traditional method to screen inhibitors of an enzyme usually involves both phytochemical isolation of pure compounds, and subsequent bioactivity assays using the corresponding enzyme, which is both labor-intensive and time/material -consuming. Later, bio-affinity methods based on the interaction between the ligands and the active sites of enzymes were developed (Zhang et al., 2004), and a further combination of bio-affinity methods (such as UF) with HPLC-MS techniques could further offer vital insight into biomolecule structures and ligand-receptor binding properties (Zhou et al., 2011; Qin et al., 2015). If one compound in the constituents of a mixture is able to interact with a specific enzyme, such as Topo I, the HPLC peak area of the binding compound will significantly increase in the HPLC chromatogram after its desorption from the binding enzyme. In this way, UF-HPLC-MS assay could be applied to screen and identify the ligand-receptor complexes from unbound compounds by directly comparing the chromatogram peak areas between activated and inactivated Topo I after ultrafiltration.

*Rhamnus davurica* has long been used as a kind of folk medicines in many Asian countries. Phytochemical investigations have found that the major constituents of the *Rhamnus* species are flavonoids, anthraquinones, naphthols, anthrone, triterpenes and their glycosides (Berhanu and Martin, 1995; Mohamed et al., 1999; Mai et al., 2001; Sharp et al., 2001; Chen et al., 2016a). The anti-proliferative assays showed that the *R.d*-60 fraction showed significantly higher inhibitory rates against SGC-7901 and HT-29, compared with the *R.d*-30 fraction. The efficacy of TCM usually depended on the characteristic of its chemical components. In order to further clarify and analyze the active compounds of *R.d*-60, ultrafiltration with Topo I coupled to HPLC-ESI-MS/MS analysis was developed and detailed afterward.

The ultrafiltration-HPLC analysis revealed that eight compounds from *R.d*-60 fraction showed specific binding toward Topo I. With the highest bio-affinity of the compound 5, the eight compounds with discrepant EFs disclosed competitively distinguished interactions with Topo I. To this end, these eight compounds were further identified or characterized by both their HPLC retention time and MS/MS spectra, and six of which, namely apigenin, quercetin, rhamnocitrin, sakuranetin, questin, and physcion, showed more potential antitumor activity with higher EFs.

### Validation of the Screening Method and the Potential Anti-proliferative Effects of the Representative Bioactive Compounds

Flavonoids are ubiquitously occurring and well-documented for their anticancer activities. To date, more than 8,000 natural flavonoids have been identified with diversified activities, and their discrepant biological activities are mainly due to their structural diversity and various chemical modifications (Malla et al., 2012). In this study, the two representative compounds, apigenin and quercetin, exerted notably inhibition to proliferation of all of the three cancer cells tested in **Table 2**.

Since Topo I relaxes supercoils by reversibly nicking duplex DNA to control DNA replication (Kim et al., 2011), hypothesis is that the two compounds could reversibly block Topo I -mediated cleavage of DNA complex, ultimately leading to DNA strand breaks and activation of apoptosis (Majumdar et al., 2015). Meanwhile, as a potent inhibitor of epidermal ornithine decarboxylase for the topical application, apigenin and quercetin significantly inhibited the incidence of skin tumorigenesis initiated by 7, 12-dimethylbenz (a) anthracene (DMBA) and promoted by 12-*O*- tetradecanoylphorbol-13-acetate (TPA) in SENCAR mice (Wei et al., 1990). Depending on the different chemical structures, flavones which contain the C2-C3 double bonds have been reported to arrest human gastric cancer cells in G1 phase, while the isoflavones which lack the C2-C3 double bonds have shown a distinct block at the G2/M transition (Matsukawa et al., 1993). Casagrande and Darbon (2001) also discovered that quercetin and apigenin showed remarkable inhibitory effect on human melanoma cells (OCM-1), the mechanisms were due to the presence of a hydroxyl group at the 3′-position of the B ring of quercetin, which could arrest cell cycle in the G1 phase, while its absence in apigenin correlated to a G2 block.

### The Structure-Activity Relationships between Flavonoids and Topo I

It is well known that flavonoids are the secondary metabolites of plants and have exhibited well-documented pharmacological effects, such as anti-inflammatory, anti-mutagenic and anticancer activities (Wu et al., 2013). Generally, the structural diversity of flavonoids based on substituent groups could largely contribute to their diversified biological activities. Previous studies further revealed that the structure-activity relationships involved the following respects: (1) the oxo functional group at position 4 and a C2-C3 double bond of C ring were both required for maximal anti-proliferative activity; (2) the presence of hydroxyl groups existing at the 3′- and 4′- positions of B ring conferred the maximal inhibitory effect; (3) the 3′ -OH of the B ring might be important for the arrested cell cycle level (Casagrande and Darbon, 2001; Xiao J.B. et al., 2014; 2015). For example, apigenin and myricetin exhibited 3.8 times higher bio-affinities against γ-globulin than the naringenin and dihydromyricetin (Xiao et al., 2011a). Meanwhile, flavone, 6-hydroxyflavone, 6-methoxyflavone, and myricetin showed lower binding affinities for common human plasma proteins (CHPP) about 10.02 to 17.82 times after hydrogenation of C2-C3 double bond of C ring (Xiao et al., 2011b). Interestingly, this coincided to our data summarized in **Table 1** and **Figure 3**, which revealed the reason that those flavones of compounds 3, 4, and 5 exhibited higher EFs than the dihydroflavones of compounds 1, 2, and 6, and confirmed the requirement of a C2-C3 double bond of C ring for the bio-affinities between the active components and Topo I. In addition, compound 4 showed markedly inhibition to proliferation on SGC-7901 (IC_50_: 12.70 ± 0.71 μg/mL) and Hep G2 (IC_50_: 4.78 ± 0.28 μg/mL), which implied that compound 4 could be a better Topo I inhibitor. A similar study also reported that quercetin significantly inhibited the cell proliferation of the gastric and colon cancer cells, as well as in leukemic cells during the cell cycle at the G1/S transition (Yoshida et al., 1992). In consideration of the above results, the successful screening and validation of these bioactive components from *R.d*-60 demonstrated the UF-HPLC-MS could be an effective method to rapidly screen and identify bioactive compounds from the complex natural products in order to discovery better natural inhibitors against Topo I and more potential natural anti-cancer candidates.

## Conclusion

In the study, a simple and rapid method using UF-HPLC-MS has been developed to screen and identify Topo I inhibitors from *R. davurica*. A total of eight components were revealed as potential Topo I inhibitors and their chemical structures were subsequently identified. The EFs of each compound screened in the *R.d-60* fraction were calculated and demonstrated to be characteristic for their binding affinity to Topo I. The antiproliferative assays of two representative ligands of interest (compounds 3 and 4) against three cancer cell lines of SGC-7901, HT-29, and Hep G2 in an intuitive dose-dependent manner further confirmed the anti-proliferative activity of those compounds fished out using UF-HPLC-MS, the reliability of this bio-affinity based screening method has thus shown good promise for the discovery and development of new natural inhibitors of target enzymes of interest. On the other hand, the structure-activity relationships revealed that flavones which contain a C2-C3 double bond at C ring exhibited higher bio-affinities between the active components and Topo I than dihydroflavones which lack the C2-C3 double bond. For the first time, the UF-HPLC-MS method was applied to screening and identifying Topo I inhibitors from *R. davurica* to the best of our knowledge, and proved to be an efficient and alternative approach for rapidly screening and identifying natural-origin Topo I ligands from natural medicinal plants.

## Author Contributions

MG conceived, designed and supervised the study. GC performed the experiments, analyzed the data, and wrote the manuscript. All authors approved and reviewed the final manuscript.

## Conflict of Interest Statement

The authors declare that the research was conducted in the absence of any commercial or financial relationships that could be construed as a potential conflict of interest.
